# A DNA break inducer activates the anticodon nuclease RloC and the adaptive immunity in *Acinetobacter baylyi* ADP1

**DOI:** 10.1093/nar/gkt851

**Published:** 2013-09-20

**Authors:** Daniel Klaiman, Emmanuelle Steinfels-Kohn, Gabriel Kaufmann

**Affiliations:** Department of Biochemistry and Molecular Biology, Tel Aviv University, Tel Aviv 69978, Israel

## Abstract

Double-stranded DNA breaks (DSB) cause bacteria to augment expression of DNA repair and various stress response proteins. A puzzling exception educes the anticodon nuclease (ACNase) RloC, which resembles the DSB responder Rad50 and the antiviral, translation-disabling ACNase PrrC. While PrrC's ACNase is regulated by a DNA restriction-modification (R-M) protein and a phage anti-DNA restriction peptide, RloC has an internal ACNase switch comprising a putative DSB sensor and coupled ATPase. Further exploration of RloC's controls revealed, first, that its ACNase is stabilized by the activating DNA and hydrolysed nucleotide. Second, DSB inducers activated RloC's ACNase in heterologous contexts as well as in a natural host, even when R-M deficient. Third, the DSB-induced activation of the indigenous RloC led to partial and temporary disruption of tRNA^Glu^ and tRNA^Gln^. Lastly, accumulation of CRISPR-derived RNA that occurred in parallel raises the possibility that the adaptive immunity and RloC provide the genotoxicated host with complementary protection from impending infections.

## INTRODUCTION

RloC is a bacterial anticodon nuclease (ACNase) (1,2) resembling the universal dsDNA break (DSB) response/repair protein Rad50 (3–6) and the translation-disabling, phage-excluding ACNase PrrC (7). These features have portrayed RloC as a potential DSB-responsive translation disabler. As such, RloC could impair the supply of needed DNA repair and stress response proteins (8). Therefore, RloC has been further proposed to function as an antiviral contingency when type I DNA restriction is alleviated under genotoxic stress (1,2,9–11). Evaluating RloC's salient traits and purported role necessitates prior account of its putative forerunner PrrC and relevant properties of Rad50.

PrrC's best characterized ortholog was encountered in a rare *Escherichia coli* strain restrictive to phage T4 mutants lacking 3′-phosphatase/5′-polynucleotide kinase (Pnk) and/or RNA ligase 1 (Rnl1) (12). PrrC's ACNase is normally silenced in this host by an associated type Ic DNA restriction-modification (R-M) protein (13,14) and turned on by a T4-encoded anti-DNA restriction peptide (15). The resulting incision of the tRNA^Lys^ anticodon loop could disable T4 late protein synthesis and contain the infection but T4's tRNA repair proteins Pnk and Rnl1 reverse the damage (16,17). This host/phage survival cascade is probably shared by most PrrC-encoding bacteria. Namely, *prrC* is invariably linked to a type Ic R-M (*hsd*) locus. Moreover, PrrC orthologs looked at exhibited ACNase activity unless linked to a cryptic R-M system (1,7,18,19). The notion that Pnk and Rnl1 evolved as ACNase antidotes (20,21) is supported by their ubiquity among T4-like phage of PrrC-encoding bacterial clades but absence from T4-like cyanophages that are not expected to encounter an ACNase (2). Pnk also figures in mRNA decay during phage T4 development (22).

PrrC's ABC-ATPase N-domain mediates the activation of the C-proximal ACNase by hydrolysing GTP and stabilizes the active ACNase by avid binding of dTTP, which accumulates in the T4-infected cell (7,23). This dual nucleotide specificity sets PrrC apart from other ABC-ATPase containing proteins, including RloC. PrrC's C-domain harbours residues implicated in tRNA^Lys^ recognition (24–27) and a putative catalytic ACNase triad (7) shared by RloC (1). PrrC is thought to act as a dimer of dimers whose C-domains dimerize in parallel and the N-domains head-to-tail as do typical ABC-ATPases (7,27). Another view is that PrrC is a dimer whose ACNase domains do not interface (18).

RloC shares PrrC's overall organization into ATPase and ACNase domains but differs in several key respects. First, *rloC* is only rarely linked to an R-M system although *in trans* interactions of non-linked orthologs have not been excluded (1). Second, isolated PrrC has overt ACNase activity indifferent to nucleotide hydrolysis but highly unstable without dTTP (7,28). In contrast, purified RloC encoded by the thermophile *Geobacillus kaustophilus* (*Gka*RloC) exhibits ACNase activity only when turned on by its ATPase in the presence of linear DNA. Once activated, *Gka*RloC's ACNase can be stabilized by the non-hydrolysable ATP analogue AMPPNP but not by dTTP (2). It has been proposed that the advent of RloC's internal ACNase switch rendered unnecessary the reliance on an external R-M silencing partner. This, in turn, could facilitate RloC's lateral gene transfer and account for its broader distribution among bacteria, compared with PrrC (2). Third, while PrrC only incises its tRNA substrate, *Gka*RloC excises the wobble nucleotide, a lesion expected to encumber phage-induced tRNA repair and likewise contribute to RloC's broader distribution (1). Fourth, a coiled-coil/zinc-hook insert (CC/ZH) likens RloC's ATPase domain to the universal DSB sensor/effector Rad50 (5). The CC/ZH stretch of Rad50 folds back into an antiparallel coiled-coil bundle and this structure protrudes from the ATPase head-domain with the ZH motif Cys-X-X-Cys at its apex. Two ZH apices join by coordinating Zn^++^ to the four Cys residues of the dimerization interface. Such joints may form within a free Rad50 dimer or between two DSB-bridging dimers. In the latter state, Rad50 directs the associated Mre11 DNase to initiate DNA end resection, a key step in DSB repair (29–32). A regulatory role of *Gka*RloC's CC/ZH is suggested by ZH mutations that uncouple its ACNase and DNA-dependent ATPase and the activation of this ACNase by linear but not circular DNA (1,2).

Further exploration of RloC's regulation reported here revealed the following. First, once turned on, RloC's ACNase is stabilized by its activating DNA and the nucleotide it hydrolysed. Second, exposure to a DSB inducer activates RloC's ACNase both in heterologous contexts and within a natural host, even when R-M mutated. Third, tRNA^Glu^ and tRNA^Gln^ are natural RloC substrates. Lastly, the DSB-induced activation of the indigenous ACNase was accompanied by accumulation of CRISPR-derived RNA (crRNA) (33), hinting that RloC can complement the adaptive immunity in defending the genotoxicated host from impending infections.

## MATERIALS AND METHODS

### Materials

Mitomycin C (MMC), nalidixic acid (NAL), serine hydroxamate (SH), nucleotide solutions, DNA oligonucleotides and 1-ethyl-3-(3-dimethylaminopropyl) carbodiimide (EDC) were purchased from Sigma; RNA oligonucleotides were obtained from Integrated DNA Technologies; [γ-^32^P]ATP from PerkinElmer; DNA restriction nucleases, DNA polymerases, T4 DNA ligase, T4 polynucleotide kinase, T4 RNA ligase 1, calf intestinal alkaline phosphatase, DNase I and pUC19 DNA from New England Biolabs; nuclease P1 from USB; RNases T1 and BC from P-L Biochemicals and a His_6_ tag antibody from Roche.

### Bacterial strains and plasmids

*Acinetobacter baylyi* ADP1 was obtained from Eliora Z. Ron, Tel Aviv University, and the *ΔrloC*, *ΔhsdR*, *ΔhsdM* and *ΔhsdS* alleles of this strain were obtained from Veronique de Berardinis, Genoscope. Expression plasmids encoding wild-type *Gka*RloC pGkaRloC-L-His_6_ and its ACNase-null derivative E696A were previously described (1).

### Plasmid construction and expression of RloC proteins

*Acinetobacter baylyi* ADP1 orf 0152 encoding the 826aa RloC protein (NCBI accession YP_044948) was amplified by polymerase chain reaction (PCR) from genomic DNA using *Pfu* DNA polymerase (Stratagene). The PCR primers used introduced an NdeI restriction site at the start codon and an AgeI site at the C-end to fuse the orf via a flexible linker to a His_6_ tag, as in plasmid pGkaRloC-L-His6 (1). To this end, the NdeI- and AgeI-digested PCR product replaced the *Gka*RloC orf of pGkaRloC-L-His6 to yield pAbaRloC-L-His6. Point mutations were introduced by Quick Change (34) and the *Gka*RloC Δ152–478 deletion created by SLIM (35). The RloC constructs were transformed into *E. coli* XL-Blue 1 (Stratagene) and then into *E. coli* RosettaTM (DE3)pLys (Novagen). The latter transformants were grown at 37°C to 0.6 OD_600_ in Luria–Bertani (LB) medium containing 100 µg ml^−^^1^ ampicilin and 34 µg ml^−^^1^ chloramphenicol. ACNase protein expression was induced by 0.05–0.1 mM isopropyl-β-d-thiogalacto-pyranoside (IPTG). Wild-type and Δ152–478 *Gka*RloC proteins were isolated as described (2).

### ACNase assays

**Figure 1.**
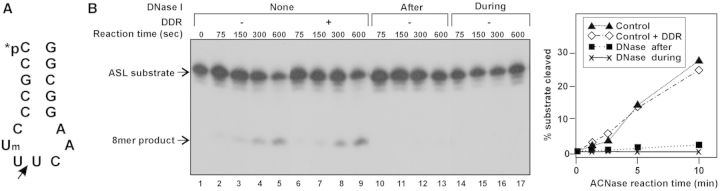
*Gka*RloC's ACNase activity is sustained by the activating DNA. (**A**) The 5′-^32-^P labelled ACNase substrate 7-2'-Om-Glu-ASL. (**B**) *Gka*RloC's ACNase was activated for 20 min in the presence of ATP and DNA followed by further pre-incubation with DNase buffer (lanes 1–5), or DNase I buffer containing purified oligonucleotides formed by a DNase I digestion of an activating DNA dose (lanes 6–9) or DNase I (lanes 10–13). In lanes 14–17 DNase I was included in the activation mixture. ACNase activity was subsequently assayed as detailed in ‘Materials and Methods’ section. DDR, DNase-I digest residue; After or During, DNase I added after the activation or during the activation, respectively; ASL, [5′-^32^P]7-2'-Om-Glu-ASL; 8mer, labelled cleavage product.

*In vivo* ACNase activity was monitored as follows. Cellular RNA was phenol extracted and separated by denaturing gel electrophoresis, as such or after any 5′-OH termini had been phosphorylated by T4 Pnk and [γ-^32^P]ATP (24). ACNase products were visualized then by respective ethidium bromide staining and autoradiography. The *in vitro* assays of *Gka*RloC used as a standard substrate a synthetic ASL oligoribonucleotide that matched in sequence *G. kaustophilus* tRNA^Glu^ and was 2′-O methylated 5′ to the wobble base (7-2′Om-Glu-ASL, Figure 1A). Other synthetic *in vitro* substrates were the parental unmodified Glu-ASL and a derivative 2′-O methylated 3′ to the wobble base (8-2′Om-Glu-ASL). These oligonucleotides were either [5′-^32^P] labelled or 3′-end labelled using [5′-^32^P]pCp and T4 RNA ligase 1 (36). The ACNase activation mixture (10 µl) contained 10–100 ng of the indicated *Gka*RloC allele, 5 ng/µl SmaI-linearized pUC19 DNA, 0.5 mM ATP, 10 mM MgCl_2_, 5 mM dithiothreitol, 70 mM Tris–HCl buffer, pH 7.5. Following incubation for 20 min at 25°C, 5–10 nM of the ASL substrate was added and the incubation at 25°C continued. The reaction was stopped with 2 volumes of 10 M urea, 0.025% each of xylene cyanoll and bromphenol blue. The products were separated by denaturing gel electrophoresis and autoradiographed.

### Identification of NAL-induced RNA fragments

*Acinetobacter baylyi* ADP1 culture in LB medium containing 10 µg/ml ampicilin and 3.4 µg/ml chloramphenicol was grown by shaking at 25–30°C. At a density of 0.6 OD, the culture was supplemented with NAL at 30 µg/ml and the incubation continued at 25°C for 4 h. The cells were harvested then, their RNA phenol extracted and any 5′-OH cleavage termini in it labelled at 3000 Ci/mmol using T4 Pnk and [γ-32 P] ATP. Following separation by denaturing gel electrophoresis, the labelled products were extracted and partially digested by mild alkaline hydrolysis or by the respective G or pyrimidine-specific RNases T1 and BC. The digests were separated by denaturing gel electrophoresis and autoradiographed, essentially as described (37). Labelled 5′-end groups released by nuclease P1 digestion were identified by 2D thin layer chromatography (38). The assumed identities of the RNA products of interest were ascertained by splint ligation essentially as described (39). Briefly, scaled up RNA preparations were weakly 5′-end labelled (1 Ci/mmol) to identify and isolate the species of interest. In the case of the RloC-independent product X suspected to be crRNA, the Pnk used for labelling also removed 3′ cyclic phosphate (40) that would prevent ligation to the strongly labelled (3000 Ci/mmol) DNA oligonucleotide probe. Bridging oligonucleotide templates used to juxtapose the ligation partners matched the predicted 3′-proximal sequence of the RNA and the sequence of the probe. After T4 DNA ligase-mediated ligation any free 5′-P groups were removed by calf intestinal alkaline phosphatase. The bridging templates and ligation oligonucleotide probe used are listed in Supplementary Table S1. Northern analysis of RNA extracted at different NAL exposure times entailed prior phosphorylation of 5′-OH to enable EDC-mediated covalent linkage to the nylon membrane (41). The blot was probed with a 5′-^32^P labelled oligonucleotide matching *A. baylyi* ADP1 tRNA^Glu^ residues 35–72 (Supplementary Table S1).

## RESULTS

### *Gka*RloC's ACNase is stabilized by its activating DNA and hydrolysed nucleotide

Thermal stability has rendered *Gka*RloC a convenient *in vitro* paradigm, superior to mesophilic orthologs. Previous data revealed that the activation of its ACNase by ATP hydrolysis requires the presence of linear DNA (2). To determine if the requisite DNA also stabilizes the activated ACNase it was degraded after the activation by DNase I. This inhibited the ACNase almost as severely as adding DNase I ahead of the activation (Figure 1B, lanes 2–5, 10–13, 14–17). To determine if this inhibition was due to the lack of the DNA or formation of the DNA degradation products we added to the activated ACNase a DNase I-degraded activating DNA dose. This addition had no effect (lanes 6–9), suggesting that continued ACNase activity required that *Gka*RloC remain tethered to the activating DNA. The ACNase substrate used 7–2′-Om-Glu-ASL (Figure 7A) is a synthetic anticodon-stem-loop matching in sequence *G. kaustophilus* tRNA^Glu^ and containing a 2′-O methyl group intended to prevent the excising cleavage 5′ to the wobble base. Using it simplified the determination of the incision rate. Surprisingly, the parental unmodified Glu-ASL was incised mainly 5′ to the wobble base, yielding a product similar to that formed exclusively with a derivative whose 3′ linkage was protected (8-2′-Om-Glu-ASL). The preferential 5′ incision occurred whether Glu-ASL was labelled at the 5′- or 3′-end (Supplementary Figure S1A and B). Because *Gka*RloC substrates with natural wobble base modifications are successively cleaved 3′ and then 5′ to the wobble base (1,2), the skipping of Glu-ASL's 3′ linkage suggested that the modified wobble base figures in *Gka*RloC's cleavage site specificity.

**Figure 2.**
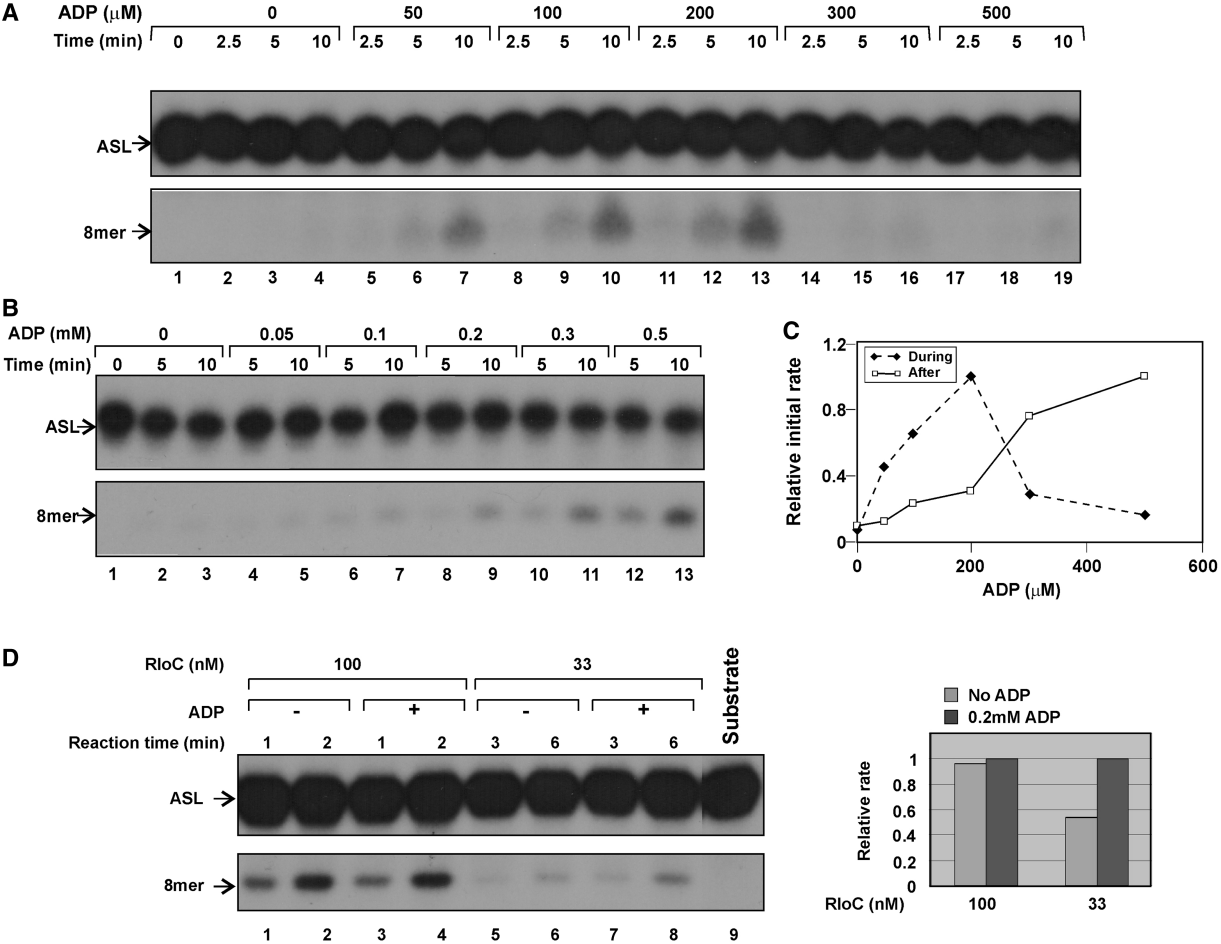
ADP stabilizes *Gka*RloC's activated ACNase. (**A**) ACNase activation mixtures were supplemented with the indicated amount of ADP and the ACNase assayed using 7-2'-Om-Glu-ASL as a substrate. (**B**) ADP was added at the indicated amounts after the activation and the ACNase assayed as in A. (**C**) Initial ACNase reaction rate versus ADP level in the activation mixture (During) or added after the activation (After). (**D**) ACNase was assayed at the indicated *Gka*RloC levels followed by activation without or with 0.2 mM ADP.

Mutating critical ABC-ATPase residues abolishes *Gka*RloC's ACNase activity. Yet, the anticipated DNA-dependent ATPase activity of wild-type *Gka*RloC could not be detected over the mutant background. This discrepancy and ability of AMPPNP to both inhibit the activation and stabilize the activated ACNase have suggested that the activation entails a single cycle of ATP binding and hydrolysis (2). To determine if AMPPNP emulates in its protective effect the nucleotide hydrolysed during the activation, ADP was included in the ACNase activation mixture or added after the activation. This protected the ACNase in either case (Figure 2A and B), but in the first, the protection sharply declined above ∼200 µM ADP, whereas in the second, it kept increasing (Figure 2C). This difference was ascribed to an inhibitory ADP/ATP competition during the activation and ability of ADP to stabilize the activated ACNase, similar to AMPPNP. ADP's protective effect became apparent only after sufficient dilution of the ACNase protein (Figure 2D). This signified perhaps synergistic effects of *Gka*RloC's oligomerization and the tethered DNA on the tendency of the hydrolysed nucleotide to dissociate from the protein.

### DSB inducers activate RloC ACNases in a heterologous context

**Figure 3.**
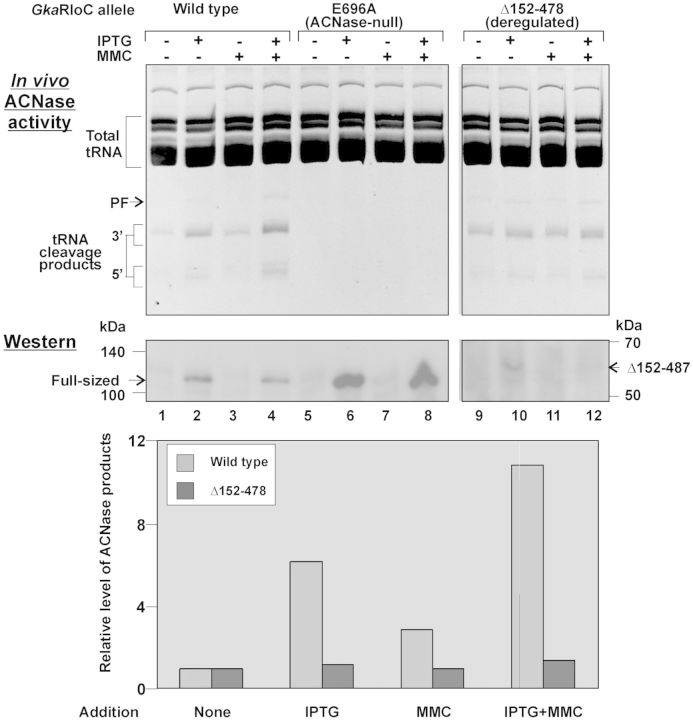
MMC activates *Gka*RloC's ACNase in *E. coli*. *Escherichia coli* Rosetta cells transformed with expression plasmids encoding the indicated *Gka*RloC alleles were incubated in the absence or presence of 200 ng/ml MMC and/or 0.1 mM IPTG. RNA was extracted, separated by denaturing gel electrophoresis and stained with ethidium bromide. *Gka*RloC protein was monitored by western blot using an anti-His_6_ tag antibody. Full-sized, *wt* or E696A *Gka*RloC protein; PF, tRNA precursor fragment.

Because circular DNA fails to activate *Gka*RloC's ACNase, DSB have been implicated in the *in vivo* activation (2). To examine this premise, we determined if the DSB-inducing inter-strand crosslinker MMC (42) will activate plasmid-borne *Gka*RloC expressed in *E. coli* from the T7-Lac promoter. As controls served the ACNase-null mutant *Gka*RloC^E696A^ (1) and the deregulated CC-truncated mutant *Gka*RloC^Δ152^^−^^478^ that featured *in vitro* a constitutive-ACNase phenotype (Supplementary Figure S2). *In vivo* ACNase activity was monitored by extracting RNA from the untreated or MMC-treated cells before or after inducing the expression of the ACNase protein with IPTG. The extracted RNA was separated by denaturing gel electrophoresis and stained with ethidium bromide. ACNase activity was indicated by the formation of the typical tRNA fragments of ∼42 and ∼33 nt and a ∼52 nt fragment thought to originate from a tRNA precursor (1). In the absence of both IPTG and MMC, *Gka*RloC yielded a basal level of ACNase cleavage products over the ACNase-null background (Figure 3, lane 1 versus 5). Adding MMC without IPTG tripled their extent (lane 1 versus 3). A higher basal activity obtained when *Gka*RloC's expression was induced (lane 2) was also augmented by MMC, albeit, only ∼2-fold (lane 4), possibly owing to exhaustion of the tRNA substrates. The specificity of these enhancements was underscored by the indifference of *Gka*RloC^Δ152-478^ to MMC (lanes 9–12). The basal ACNase activity of wild-type *Gka*RloC is ascribed to partially degraded forms that could behave like the constitutive mutant and/or spontaneous DSB.

**Figure 4.**
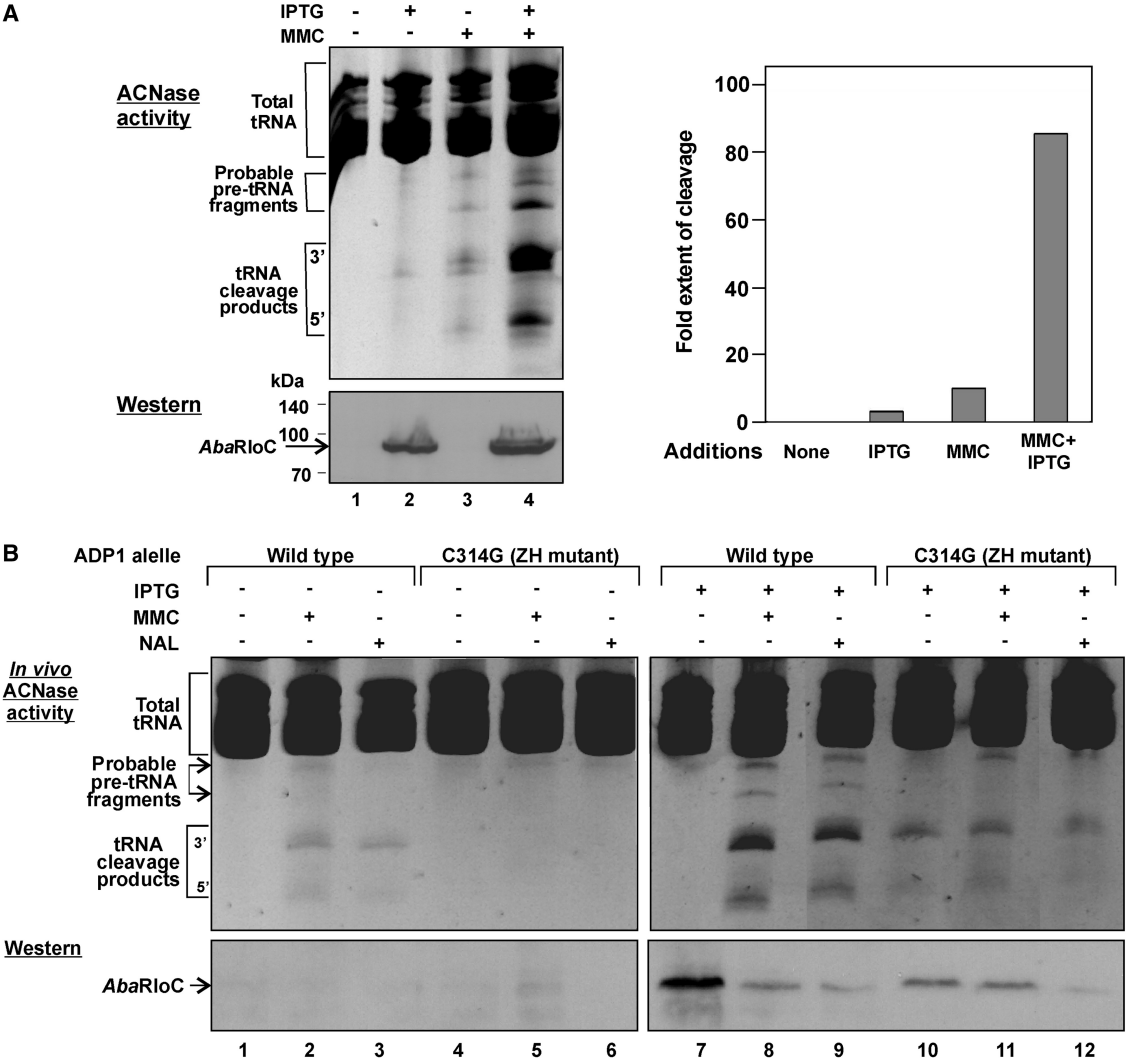
DSB inducers activate *Aba*RloC in *E. coli*. (**A**) *Escherichia coli* Rosetta cells expressing *Aba*RloC from a plasmid were incubated in the presence or absence of MMC and/or IPTG and *in vivo* ACNase activity and *Aba*RloC protein monitored as in Figure 3. (**B**) Similar to (A) except that the cells encoded the indicated *Aba*RloC alleles and were exposed also to 30 µg/ml NAL.

Weaker basal ACNase activity and a more pronounced response to MMC were observed when *Gka*RloC was replaced by the mesophilic *A. baylyi* ADP1 RloC (*Aba*RloC) (Figure 4A). In this case ACNase, cleavage products were not detected in the absence of both IPTG and MMC (lane 1). They appeared with IPTG alone (lane 2) but at a level ∼4-fold lower than with MMC alone (lane 3) and ∼30-fold lower than with both IPTG and MMC (lane 4). Another DSB-inducing agent, the DNA gyrase inhibitor NAL (43) activated *Aba*RloC's ACNase similarly (Figure. 4B, lane 2 versus 3 and 8 versus 9). In contrast, the overt ACNase of the ZH mutant *Aba*RloC^C314G^ was indifferent to either DSB inducer (lanes 10–12). This behavior could be demonstrated only when *Aba*RloC^C314G^'s expression was induced, possibly due to its weaker ACNase activity and/or stability.

### DSB-induced activation of indigenous RloC

**Figure 5.**
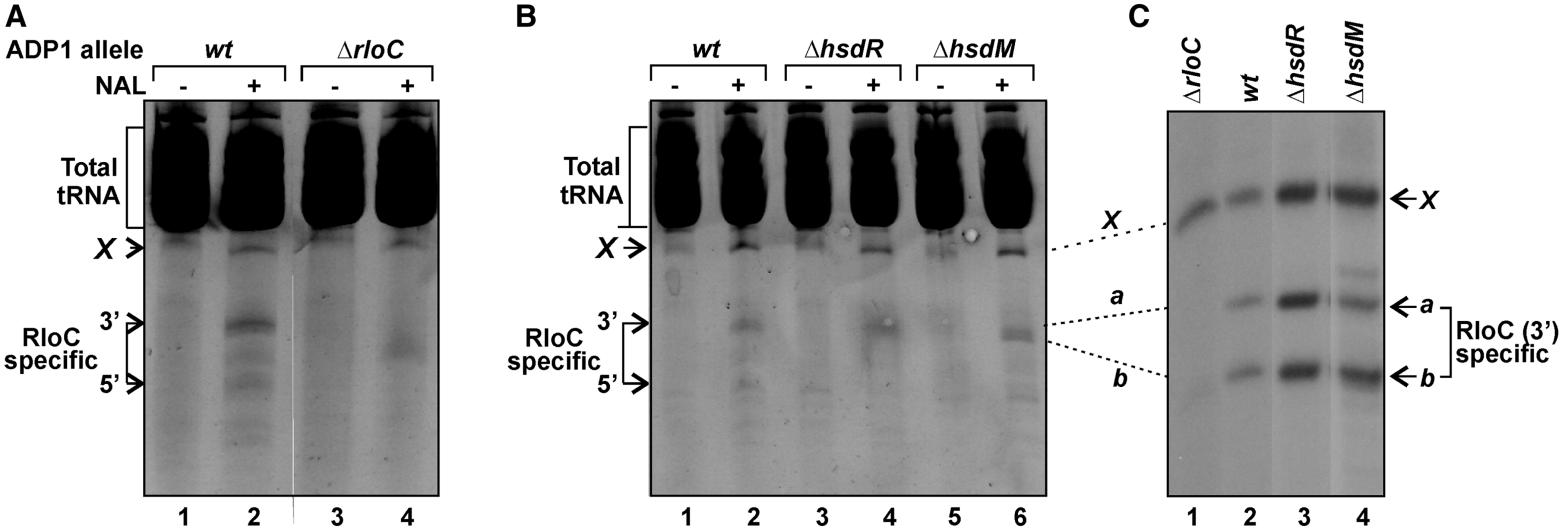
NAL activates the indigenous *Aba*RloC ACNase. (**A**) The wild-type or *ΔrloC* alleles of *A. baylyi* ADP1 were grown for 4 h at 25°C in the absence or presence of 30 µg/ml of NAL and *in vivo* ACNase activity monitored as in Figure 3. X indicates the RloC-independent product accumulating in the presence of NAL. (**B**) The indicated *A. baylyi* ADP1 alleles were examined for NAL-induced ACNase activity essentially as in panel A. (**C**) 5′-OH termini in RNA extracted from the indicated *A. baylyi* ADP1 alleles exposed to NAL were radiolabelled, the RNA separated then on a long gel in which the 3′ fragments generated by RloC were resolved in two major bands designated ‘a’ and ‘b’. The broken lines between panels B and C match the stained fragments with their 5′-end labelled counterparts.

We chose to investigate RloC's natural activation using *A. baylyi* ADP1 since this soil bacterium is easy to grow and genetically manipulate (44). Moreover, a library of single gene deletion mutants exists for all its dispensable genes (45), including *rloC* (ACIAD0152) and the non-linked *hsdR*, *hsdM* or *hsdS* genes of the respective R-M restriction, modification or specificity subunit (ACIAD343-2). However, an infectious phage is not known yet. Prolonged exposure of this host to a high MMC dose (1 µg/ml) failed to activate the indigenous RloC ACNase, consistent with its resistance to MMC (46). Unaware of this caveat we have suggested that DSB only set the indigenous *Aba*RloC for activation by an added, possibly phage-induced signal (2). However, exposing *A. baylyi* ADP1 to the toxic DSB inducer NAL sufficed to activate the indigenous RloC ACNase. This was inferred from the formation of ACNase-like cleavage products in the wild-type (Figure 5A, lanes 1 and 2) but not *ΔrloC* allele (lanes 3 and 4). Interestingly, all three R-M mutants featured the wild-type phenotype (shown in Figure 5B for *hsdR* and *hsdM*), indicating that the R-M protein neither silences *Ab*RloC's ACNase nor mediates its DSB-induced activation.

The level of the NAL-induced cleavage products formed by *Aba*RloC in its natural host relative to the total RNA was far lower than observed in the heterologous *E. coli* context, even in the absence of IPTG. This often impeded the detection of the ACNase cleavage products over the background of non-specific RNA fragments. Monitoring the indigenous ACNase activity was facilitated by 5′-end labelling, within the total RNA preparation, of the 3′ tRNA fragments the ACNase generated. Separating the labelled RNA on a long gel resolved the RloC-specific fragments into two bands designated ‘a’ and ‘b’ (Figure 5C). NAL induced in *A. baylyi* ADP1 also RloC-independent small RNA species. The most prominent of them named X was also 5′-end labelled, indicating that it originated by transesterifying cleavage of a larger species. Its identification as crRNA (33) is described in a later section.

### *Aba*RloC targets tRNA^Glu^ and tRNA^Gln^

**Figure 6.**
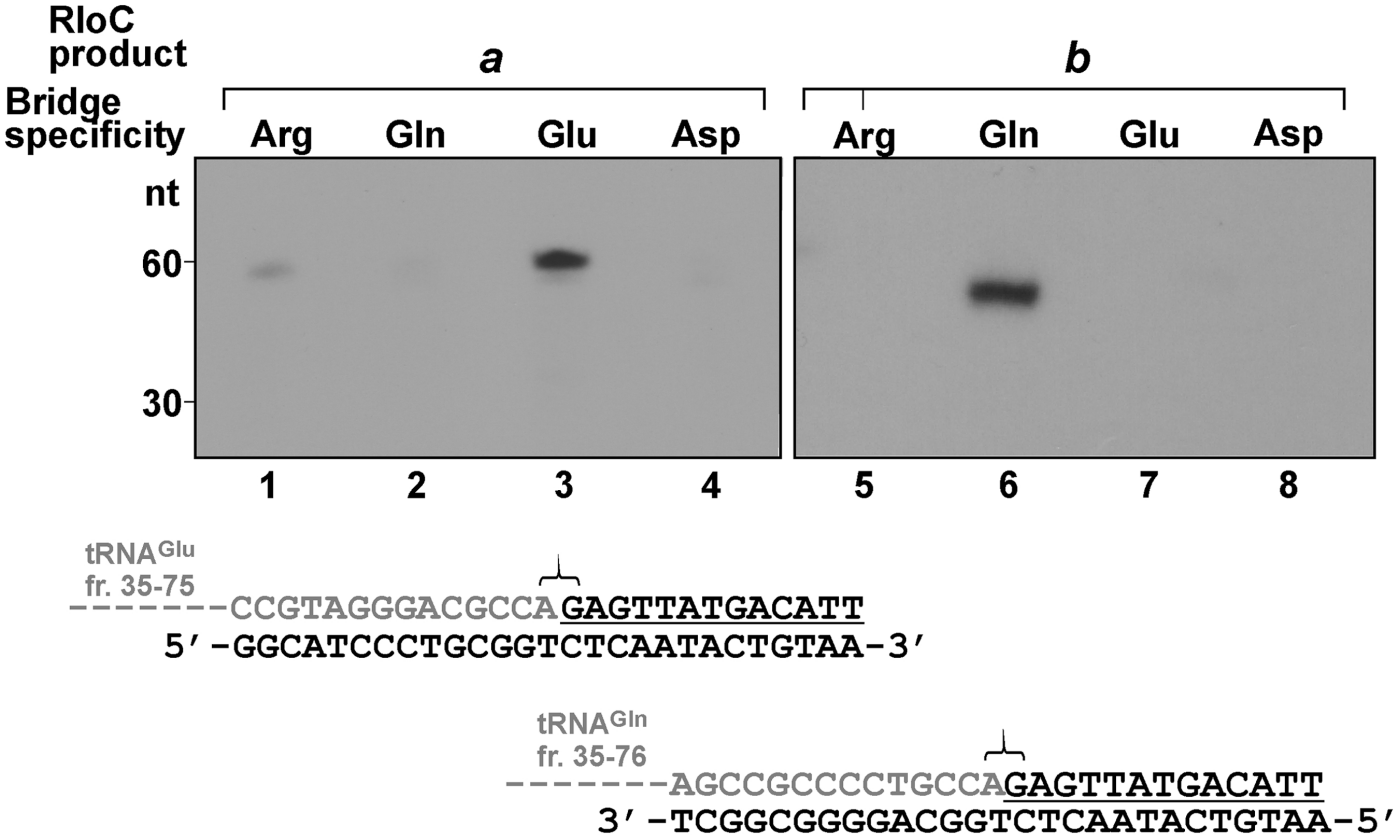
tRNA^Glu^ and tRNA^Gln^ are natural *Aba*RloC substrates. RloC-dependent products ‘a’ and ‘b’ (Figure 5C) labelled at low specific radioactivity (1 Ci/mmol) were subjected to splint ligation (39) using a ligation oligonucleotide probe 5′-labelled at high specific activity (3000 Ci/mmol) and bridging oligonucleotides complementary to 3′ portions of Arg, Gln, Glu or Asp specific *A. baylyi* ADP1 tRNAs (Supplementary Table S1). Following phosphatase treatment the ligation products were separated by denaturing gel electrophoresis and monitored by autoradiography. The splint ligation set-ups shown below identified the tRNA^Glu^ fragment ‘a’ (left) and the tRNA^Gln^ fragment ‘b’ (right). The non-specific NAL-induced product X and Xylene cyanoll were used as respective 60 and 30 nt markers.

To identify the RNA species targeted by the NAL-activated, indigenous *Aba*RloC, the 5′-^32^P-labelled fragments ‘a’ and ‘b’ (Figure 5C) were partially digested by base specific RNases. The product pattern obtained suggested that fragment ‘a’ originated from tRNA^Glu^ and fragment ‘b’ from tRNA^Gln^ (Supplementary Figure S3A and B). Complete digestion of either with nuclease P1 released mainly labelled pU (Supplementary Figure S3C), commensurate with RloC-mediated incision of the original tRNA species 3′ to the wobble base. The tRNA^Glu^ and tRNA^Gln^ assignments were ascertained then by splint ligation (39) using weakly labelled (1 Ci/mmol) fragments ‘a’ or ‘b’ and a strongly labelled ligation partner (3000 Ci/mmol). Ligation of fragment ‘a’ to this probe was enabled by a bridging oligonucleotides matching a 3′ proximal portion of tRNA^Glu^ (Figure 6, lane 3) but not by counterparts matching Asp or Gln specific tRNAs. The weak signal obtained with the tRNA^Arg^ specific probe could reflect some cross hybridization due to shared sequences but leaves open the possibility that this species was also cleaved. Fragment ‘b’ was ligated to the probe only with the tRNA^Gln^ specific bridge (lane 6).

### Dynamics of RNA cleavage products induced by NAL in *A. baylyi* ADP1

**Figure 7.**
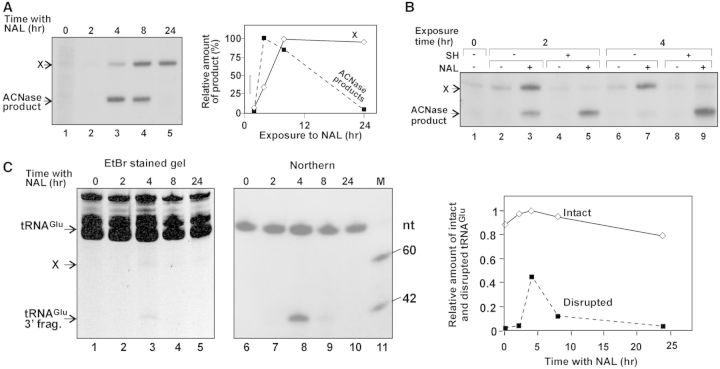
Dynamics of RNA species induced by NAL in *A. baylyi* ADP1. (**A**) Time course of formation and disappearance of the NAL-induced RNA products. RNA extracted from *A. baylyi* ADP1 at the indicated times of exposure to 30 µg/ml NAL was 5′-end labelled, separated by denaturing gel electrophoresis and autoradiographed. (**B**) NAL activates pre-existing *Aba*RloC. The dynamics of RNA cleavage products induced by 60 µg/ml NAL were followed essentially as in A in the absence or presence of 100 µg/ml SH. (**C**) The NAL-activated indigenous *Aba*RloC does not deplete its tRNA target. RNA extracted from *A. baylyi* ADP1 at the indicated times of exposure to 30 µg/ml NAL was separated by denaturing gel electrophoresis, stained with EtBr (left panel) and subjected to northern blot analysis using a 5′-labelled DNA probe complementary to tRNA^Glu^ residues 35–72 (right panel). Product X and 3′ tRNA fragments generated by *Aba*RloC were the respective 60 and 42 nt markers.

The dynamics of 5′-OH containing RNA fragments formed during *A. baylyi* ADP1's exposure to NAL were followed by monitoring the appearance and eventual decay of their *in vitro* labelled derivatives. The RloC specific fragments were detected in a logarithmic culture grown at 25°C only ∼2 h after the exposure to 30 µg/ml NAL. They reached peak intensity after ∼4 h and declined during longer incubation. In contrast, the RloC-independent product X accumulated (Figure 7A). To determine if the formation of any of these products required new protein synthesis the culture was exposed to both NAL and the seryl-tRNA synthase inhibitor and stringent response inducer SH (47). SH did not prevent the NAL-induced activation of *Aba*RloC's ACNase, causing rather the accumulation of its cleavage products (Figure 7B). This accumulation could be ascribed to enhancement of *Aba*RloC's activation due to exacerbated DNA damage in the absence of newly synthesized DNA repair proteins. Alternatively, or in addition the accumulation of the ACNase products could be due to the lack of an RNase responsible for their further decay. On the other hand, the accumulation of X was severely inhibited by SH (Figure7B). Thus, NAL must have activated pre-existing *Aba*RloC molecules, while the enhanced production of X and perhaps also the decay RloC's cleavage products depended on new protein synthesis. It is also noteworthy that the NAL dose used in this experiment was 60 µg/ml, 2-fold higher than the standard. This increase hastened *Aba*RloC's activation and decay of its cleavage products as well as the accumulation of X (Figure 7B), possibly owing to exacerbation of the DNA damage. However, SH exerted the same effects at either NAL level (not shown).

Northern analysis of RNA samples extracted during an extended exposure to NAL suggested that the activated *Aba*RloC cleaved only a minor fraction of its tRNA targets. As shown, at 4 h of exposure to 30 µg/ml NAL the peak signal of the 3′ fragment of tRNA^Glu^ amounted only to 40% of the intact tRNA^Glu^ signal (Figure 7C, lane 8). Moreover, during prolonged incubation the fragment signal sharply declined while that of the intact tRNA hardly changed (lanes 9 and 10). The true proportion of the cleavage product could be yet smaller because it was expected to hybridize to the probe more efficiently than the self-folding intact tRNA. Thus, the NAL-activated indigenous ACNase either partly disrupted its targets population-wide or depleted them in only a minor fraction of the cell population.

### Identification of X

**Figure 8.**
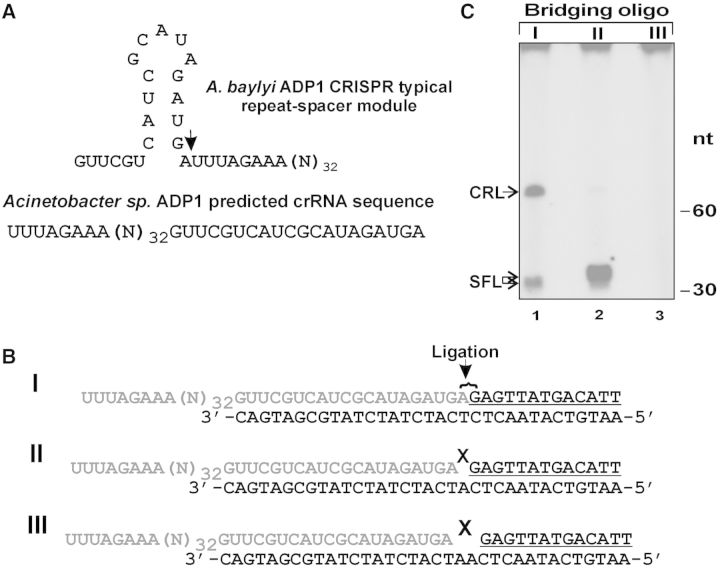
X comprises crRNA molecules. (**A**) CRISPR repeat-spacer unit of *A. baylyi* ADP1 (top) and the expected crRNA (bottom). The expected site of Cascade-mediated cleavage of the repeat is indicated by the arrow. (**B**) Splint ligation set-ups. crRNA (gray) and the labelled ligation oligonucleotide probe (underlined) are juxtaposed by a bridging oligonucleotide matching the 3′ proximal repeat portion of crRNA and the ligation partner (Set-up I). The brace sign (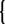) indicates that ligation is expected. In the control Set-ups II and III the bridging oligonucleotide contains one or two extra A residues expected to preclude ligation (indicated by X). (**C**) Ligation products were separated by denaturing gel electrophoresis and monitored by autoradiography. crRNA (X) and xylene cyanoll were respective 60 and 30 nt markers. CRL–crRNA ligated to the probe, SFL–self-folded bridges ligated to the probe.

Partial cleavage by RNase T1 suggested that X comprises a heterogeneous population sharing 3′ and possibly 5′ proximal sequences but highly variable internally (Supplementary Figure S3D). This pattern, an apparently uniform size of ∼60 nt and a shared 5′-OH containing terminal U (Supplementary Figure S3C) suggested that X comprises crRNA molecules (40,48). This assumption was confirmed by splint ligation where weakly labelled X devoid of any 3′ cyclic-P (40) was probed with the strongly labelled ligation partner juxtaposed by a bridging template matching also the 3′ repeat portion of *A. baylyi* ADP1 crRNA (Figure 8B, Set-up I). Following T4 DNA ligase-mediated ligation and phosphatase treatment to remove remaining 5′-P groups, Set-up I yielded a ligation product migrating as would X extended by the 13-nt ligation partner (Figure 8C, lane 1). Thus, X could be derived from a CRISPR transcript by canonical transesterifying cleavages 3′ to U_8_ of the 28-nt repeats. X was not ligated to the probe in the control Set-ups II and III (lanes 2 and 3) where the bridging templates matched ‘mock’ crRNAs resulting from Cascade-mediated cleavage 1 or 2 positions 3′ to the canonical site (lanes 2 and 3). The small ligation products seen near the 30-nt marker in lanes 1 and 2 could be derived from self-folded forms of the respective bridging templates.

## DISCUSSION

The resemblance of RloC to Rad50 and PrrC and characterized properties of one of RloC's orthologs have portrayed this ACNase as a potential DSB-responsive translation disabler (1,2). The foregoing data concur with this idea: heterologously expressed RloC ACNases were activated by DSB inducers to an extent that limited their own expression (Figures 3 and 4), possibly through depletion of targeted tRNA species and consequent translation arrest. A DSB inducer activated RloC's ACNase also in a natural host (Figure 5). However, in this case, only a minor fraction of the targeted tRNAs was disrupted (Figure 7). Whether this outcome reflected the partial cleavage of these tRNAs throughout the population or their depletion in only a minor fraction of the cells is not known. This uncertainty leads us to consider alternative ways in which RloC could benefit the genotoxicated host. Basically we consider two possibilities that need not be mutually exclusive. First, RloC benefits its host as a persistence factor augmenting tolerance to DSB inducers. Second, RloC acts as an antiviral contingency when type I DNA restriction is alleviated following DNA damage (11) and/or activated by the phage-mediated host DNA degradation. Relevant to the second task seems the possibility that RloC and the co-activated adaptive immunity (Figures 5, 7 and 8) provide the genotoxicated host with complementary defences against impending infections. These and other notions invoked by the data are discussed below.

### RloC as a DSB sensor/effector

Linear DNA was required not only for activating *Gka*RloC ACNase but also for sustaining its activity (Figure 1). Given that the linear DNA mimics DSB, the need for its continued presence portrays RloC as a sensor/effector responsive only during the lifetime of its triggering DSB signal. In other words, once activated, RloC ACNase keeps functioning as long as it remains tethered to DSB termini. Its removal with the repair of the DNA lesion prompts its inactivation, thus precluding unnecessary loss of needed tRNAs. The continued activity of RloC's ACNase seems to depend also on its ability to retain the nucleotide hydrolysed during its activation (Figure 2), a process likely to entail a single cycle of ATP binding and hydrolysis (2). Stabilization of protein on/off states by persistent binding of a hydrolysed nucleotide characterizes key cellular processes, including DNA replication initiation (49) and G protein signalling (50). In the case of RloC, the bound hydrolysed nucleotide may safeguard continued ACNase activity as long as the DSB signal persists but also elicit rapid inactivation of the ACNase when its function is no longer needed, e.g. by dissociating from the protein as the latter departs from the tethered DNA.

### RloC's natural tRNA targets

PrrC's natural specificity for tRNA^Lys^ is compromised when ectopically expressed (24). Therefore, it was not possible to tell if the preferred cleavage of tRNA^Glu^ by the ectopically expressed *Gka*RloC reflects the true substrate specificity of this ACNase (1). However, the identification of tRNA^Glu^ as a target of indigenous *Aba*RloC (Figure 6) and poor sequence identity of the two orthologs (33%) suggest tRNA^Glu^ as a common RloC target. *Aba*RloC's dual tRNA^Glu^/tRNA^Gln^ specificity suggests their shared s^2^mnm^5^U wobble base and U at the second anticodon position as RloC substrate recognition cues. However, tRNA^Lys^ that also shares these features was not detectably cleaved, pointing at the third anticodon base as a possible added cue. Specifically, C and G that occupy the third position in the targeted species have similar Watson–Crick donor–acceptor edges. This similarity may be exploited by the ACNase to counter-select the different edge of the matching U of tRNA^Lys^.

### Can RloC team with an R-M protein *in trans*?

As mentioned, a genetically linked type Ic R-M protein silences PrrC's ACNase in the uninfected cell while a phage-encoded anti-DNA restriction factor alleviates this silencing. In contrast, RloC, which is only rarely linked to an R-M protein, has an internal ACNase switch based on its DSB-responsive ATPase. This coincidence underlies a proposal that the advent of the internal ACNase switch rendered unnecessary the reliance of a PrrC-like progenitor of RloC on the external R-M regulator (2). The DSB-induced activation of *Aba*RloC in the R-M deficient mutants of its natural host (Figure 5B) concurs with this assumption. Namely, it indicates that the R-M protein neither silences RloC's ACNase nor mediates its DSB-induced activation. Nonetheless, we cannot rule out that *Ab*aRloC and other RloC orthologs not linked to an R-M protein can be still regulated by the latter in different situations such as phage infections. In favour of this possibility argues RloC's ability to excise the wobble nucleotide, as this property could have evolved to defy phage-induced tRNA repair that counteracts the milder lesion inflicted by PrrC. As T4 family members are the only phage known to encode tRNA repair (http://phage.ggc.edu/), RloC may benefit its hosts as a means to counteract this class. We consider therefore the possibility that RloC ACNases may interface an R-M protein even if not genetically linked to it. As such, they may be activated by phage anti-DNA restriction factors. However, RloC's activation during infection by a T4 family phage may be induced rather by the expected massive degradation of the host DNA, as with the T4 prototype (51). Such vigorous DNA disruption could prompt fast activation of the ACNase, sufficient to abort the infection. The hastened activation of *Aba*RloC's ACNase at the higher level of the DSB inducer or when the synthesis of DNA repair proteins was blocked by SH (Figure 7B) supports this possibility.

### How can RloC benefit the genotoxicated host?

Preliminary toxicity tests indicated that wild-type *A. baylyi* ADP1 was as sensitive to NAL as its *ΔrloC*, *ΔhsdR*, *ΔhsdM ΔhsdS* mutants. Although the *ΔrloC* mutant was less viable than wild type after prolonged incubation (regardless of NAL), the R-M mutants showed similar or even lower viability under these conditions (Supplementary Figure S4 and data not shown). We assume therefore that presence of the *tdk-kan^R^* cassette used to create all the deletion mutants is responsible for their lower viability (45). Thus, the DSB-induced activation of the indigenous ACNase may have provided the genotoxicated host with subtle or even no advantage. The possibility that RloC confers a subtle advantage such as an increasing tolerance to DSB inducers will have to be examined by suitable persistence tests. On the other hand, lack of advantage could reflect RloC's ‘imperfection’, being accidentally activated throughout the cell population, cleaving only a minor fraction of the tRNA targets in all or the majority of the cells. Such accidental activation could reflect a potential for a more vigorous activation in direr circumstances that call for cytostatic or suicidal cessation of translation, e.g. the phage-induced host DNA degradation already mentioned. RloC seems suited to ward off such phage also owing to the immediate shut-off of host gene expression their infection entails (51). The lack of host gene expression need not affect the activation of pre-existing RloC molecules but could preclude the accumulation of crRNA, which depended in *A. baylyi* ADP1 on new protein synthesis (Figure 7B).

Alternatively, the NAL-activated, indigenous *Aba*RloC could provide advantage to a minor fraction of the cell population in which the targeted tRNA species were depleted, leading to cytostatic or even suicidal translation arrest. A cytostatic response could rescue such outliers from DSB-induced lytic prophage development, while a suicidal response could prevent superinfection of sibling cells, i.e. if the prophage lacked immunity. Relevant to such scenarios may be perhaps the P2-family prophage spanning *A. baylyi* ADP1 genes 2137–2200, none of which seems to encode a homolog of a known phage immunity protein. As a lytic prophage thwarter, RloC could also make up for shortcomings of the co-activated adaptive immunity. Namely, although an artificial CRISPR construct could prevent lytic phage λ induction (52), the adaptive immunity may not be designed to do so in nature, judged by the degenerate CRISPR repeats flanking autoimmune spacers (53).

As mentioned, DNA insults cause bacteria to shut-off a key antiviral device, the type I R-M system (9). This measure prevents degradation of fully unmodified DNA synthesized during the recovery from DNA damage but at the cost of increased susceptibility to external infections (10,11). The coincident DSB-induced activations of RloC and the adaptive immunity may therefore signify their ability to provide the genotoxicated host with compensatory complementary protection from impending infections. A case of complementing antiviral defences provided by the adaptive immunity and a type II R-M system has been recently reported (54).

## SUPPLEMENTARY DATA

Supplementary Data are available at NAR Online.

## FUNDING

Israel Science Foundation [51/10 to G.K.]. Funding for open access: Israel Science Foundation.

*Conflict of interest statement*. None declared.

## Supplementary Material

Supplementary Data
