# Supplementary material for: A DNA break inducer activates the anticodon nuclease RloC and the adaptive immunity in Acinetobacter baylyi ADP1
Source: Nucleic Acids Res. 2013 Sep 20;42(1):328–39. doi: 10.1093/nar/gkt851 (PMC3874168; doi:10.1093/nar/gkt851)
Supplement: Supplementary Data [file supp_42_1_328__index.html]

A DNA break inducer activates the anticodon nuclease RloC and the adaptive immunity in Acinetobacter baylyi ADP1 — A DNA break inducer activates the anticodon nuclease RloC and the adaptive immunity in Acinetobacter baylyi ADP1 — Supplementary Data 

# A DNA break inducer activates the anticodon nuclease RloC and the adaptive immunity in *Acinetobacter baylyi* ADP1

## Supplementary Data

files

**Files in this Data Supplement:**

- Supplementary Data - pdf file
