# Supplementary material for: A DNA break inducer activates the anticodon nuclease RloC and the adaptive immunity in Acinetobacter baylyi ADP1
Source: Nucleic Acids Res. 2013 Sep 20;42(1):328–39. doi: 10.1093/nar/gkt851 (PMC3874168; doi:10.1093/nar/gkt851)
Supplement: Supplementary Data [file supp_gkt851_nar-02230-h-2013-File010.pdf]

Supplementary Table 1. DNA oligonucleotides used in splint ligation and Northern analyses

Type	Sequence[a]
**Splint ligation**	
$tRNA^{Glu}$ specific template	AATGTCATAACTCTGGCGTCCCTACGG
$tRNA^{Gln}$ specific template	AATGTCATAACTCTGGCAGGGGCGGCT
$tRNA^{Arg}$ specific template	AATGTCATAACTCTGGTGCGCTCAGAGA
$tRNA^{Asp}$ specific template	AATGTCATAACTCTGGCGCAGCGGACG
crRNA specific template	AATGTCATAACTCTCATCTATGCGATGAC
crRNA control template 1	AATGTCATAACTCATCATCTATGCGATGAC
crRNA control template 2	AATGTCATAACTCAATCATCTATGCGATGAC
Splint ligation partner	GAGTTATGACATT
**Northern**	
$tRNA^{Glu}$ specific probe	GGCGTCCCTACGGGGATTCGAACCCCGGTTACCGCCGTGA

Figure S1

A

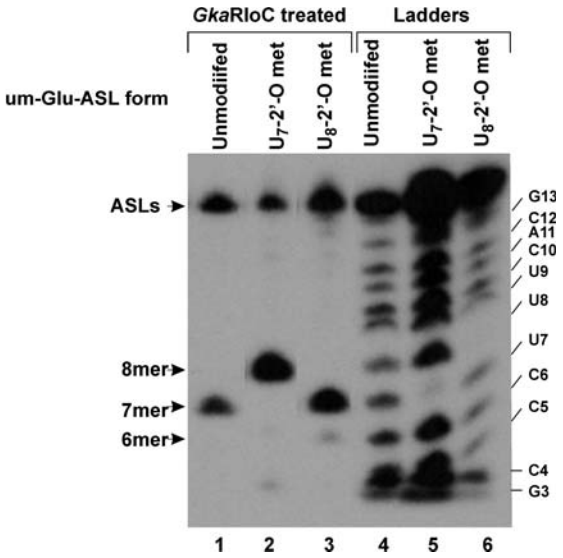

B

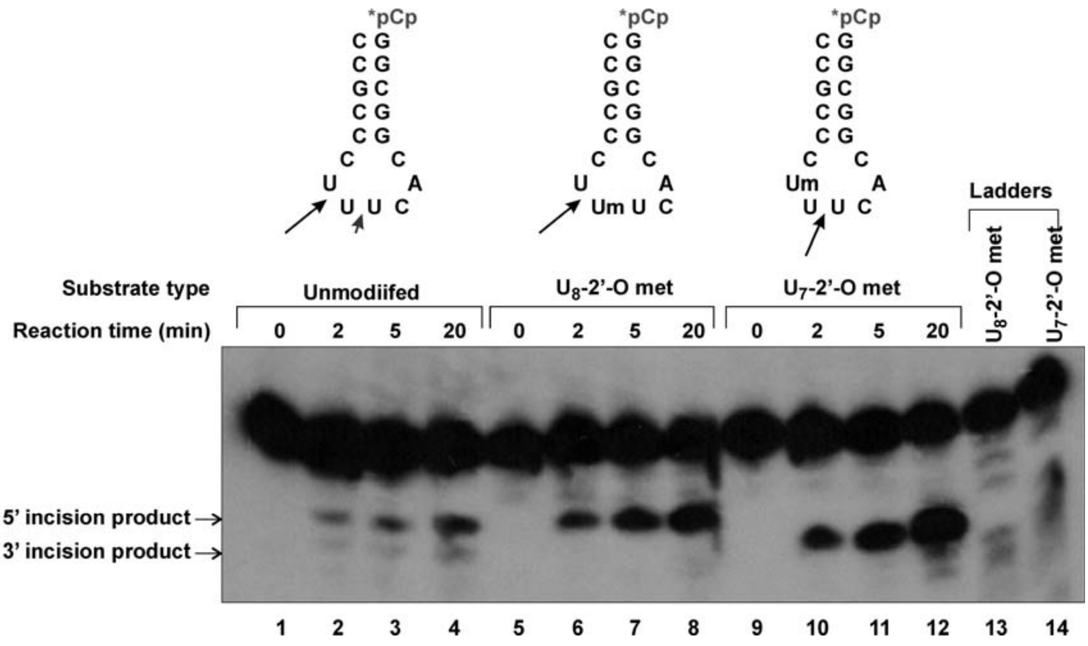

Supplementary Figure 1. *Gka*RloC skips the 3' incision site of an unmodified ASL substrate. A. Activated *Gka*RloC's ACNase was assayed as described in Methods using the [5'-^{32}P] labeled substrates Glu-ASL (lane 1) or derivatives that were 2'-O methylated 5' or 3' to the wobble base, 7 or 8-2'-Om-Glu-ASL, respectively (lanes 2 and 3, respectively). The gel was calibrated with the partially hydrolyzed ASLs (lanes 4-6). In the resultant ladders of the modified ASLs positions 7 or 8 are indicated by the respective absence of a cleavage product. B. The ASLs were as indicated in A but were 3'-end labeled by ligating to them [5'-^{32}P]pCp. Their incision sites are indicated by arrows. The unequal arrows of the unmodified substrate indicate the ~3:1 ratio of the 5' and 3' incision products.

Figure S2

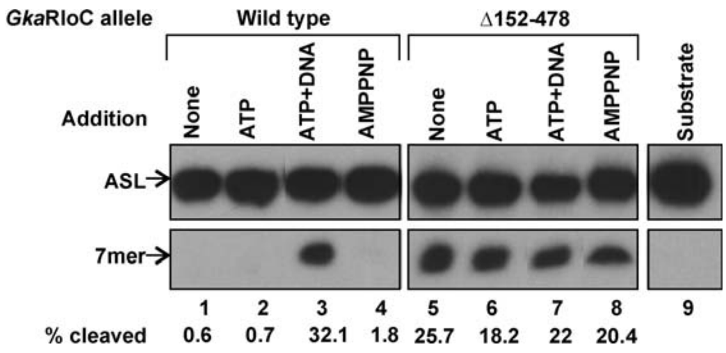

Supplementary Figure 2. The CC-truncated *Gka*RloC mutant Δ152-478 exhibits overt ACNase activity indifferent to DNA and ATP hydrolysis. The indicated *Gka*RloC alleles were assayed using an ASL substrate matching in sequence and base modification human $tRNA^{Lys3}$ in the presence of the indicated additions, essentially as described (2)**.**

Supplementary Figure S3

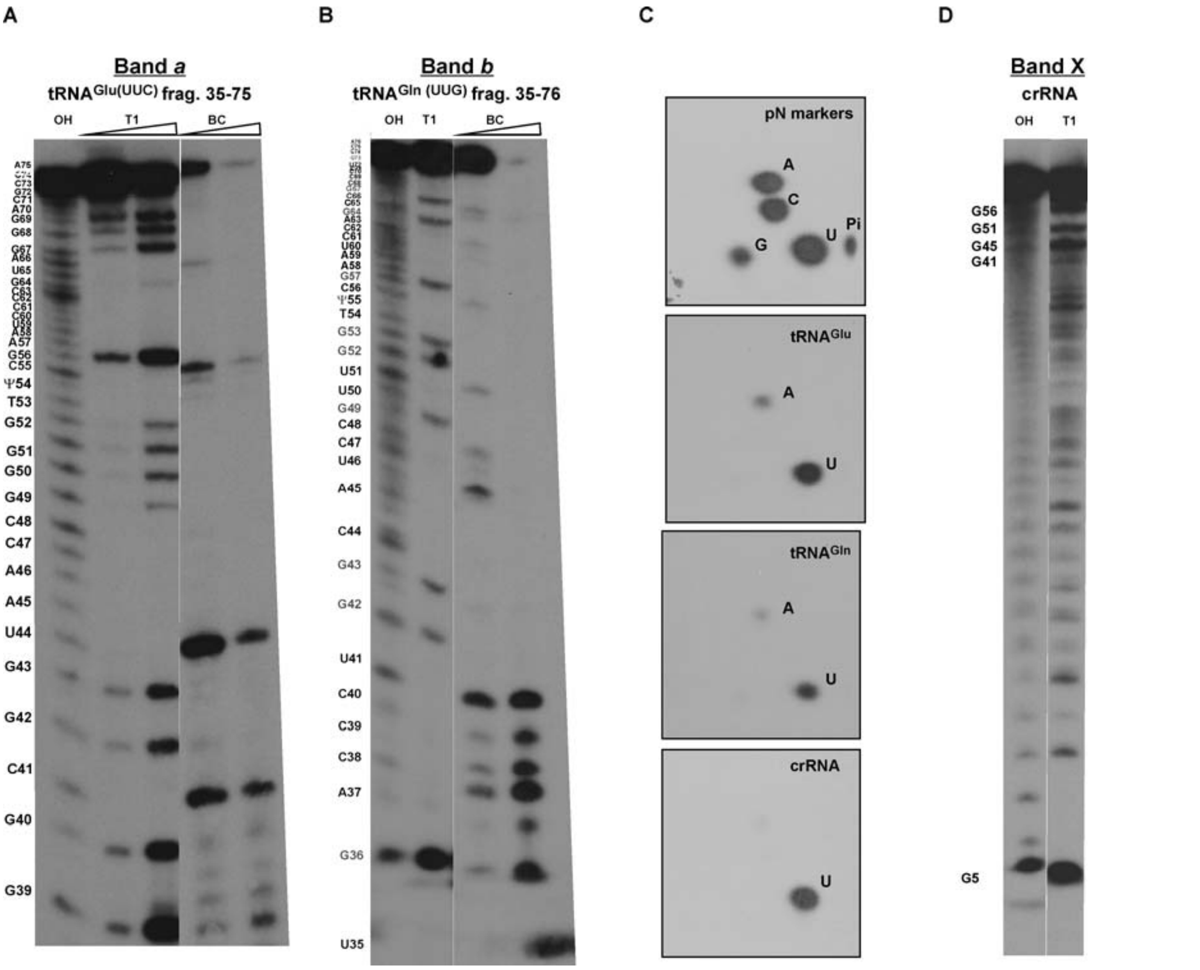

Supplementary Figure S3. Sequence analysis of the NAL induced 5'-OH containing RNA fragments formed in *A. baylyi* ADP1. A, B. The $5'$-^{32}P labeled fragments *a* and *b* (Figure 5C) were partially degraded by alkali or the respective G or pyrimidine specific RNases T1 and BC and separated by denaturing gel electrophoresis essentially as described (37). RNase T1 was employed at 10^{-4} and 10^{-3} units per 5μl reaction mixture in panel A and at 10^{-3} units in panel B. RNase BC was employed at 10^{-3} and 10^{-2} units per 5ul reaction mixture both in A and B. The sequences arrayed to the left of the auto-radiograms of panels A and B indicate the predicted sequences of the indicated 3' tRNA fragments. C. The $5'$-^{32}P fragments *a*, *b* and the RloC independent counterpart *X* identified as crRNA (Figure 8) were exhaustively digested with nuclease P1 and the products separated by two dimensional thin layer chromatography along with labeled pN markers. E. Band X was partially hydrolyzed by alkali or digested by RNase T1 (10^{-3} units). The numbered G arrayed at the left of the autoradiogram indicate the expected G residues of the 5' and 3 proximal repeat portions of A. baylyi ADP1 crRNA.

Supplementary Figure S4

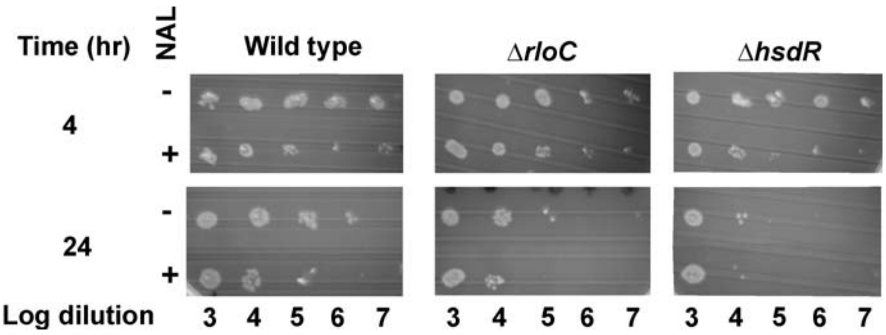

Supplementary Figure S4. NAL-toxicity assay of wild type, *ΔrloC* and *ΔhsdR* alleles of A. baylyi ADP1. The indicated alleles were grown as such or in the presence 30μg/ml NAL at 25°C for the indicated time and plated at the indicated serial dilutions on LB plates.
